# The complete mitogenome of the fairy shrimp *Streptocephalus cafer* (Lovén, 1847) (Crustacea: Branchiopoda: Anostraca) from an ephemeral pond in Botswana, southern Africa

**DOI:** 10.1080/23802359.2019.1711222

**Published:** 2020-01-14

**Authors:** Murphy Tladi, Tatenda Dalu, D. Christopher Rogers, Casper Nyamukondiwa, Shilpa Pradeep Parbhu, Peter R. Teske, Arsalan Emami-Khoyi, Ryan John Wasserman

**Affiliations:** a Department of Biological Sciences and Biotechnology, Botswana International University of Science and Technology, Palapye, Botswana;; b Department of Ecology and Resource Management, University of Venda, Thohoyandou, South Africa;; c Kansas Biological Survey and the Natural History Museum, Biodiversity Institute, Kansas University, Lawrence, Kansas, USA;; d Centre for Ecological Genomics and Wildlife Conservation, Department of Zoology, University of Johannesburg, Auckland Park, South Africa

**Keywords:** African anostracans, illumina, mitogenome, phylogenetics, Streptocephalidae

## Abstract

Fairy shrimps (Anostraca) constitute an important component of seasonally aquatic habitats, but few complete mitochondrial genomes have been published for this group. Here, we report the mitogenome of a common southern African species, *Streptocephalus cafer*, from Botswana (accession number: MN720104). Low-coverage shotgun sequencing recovered two contigs 15653 bp and 1347 bp in length that are separated by a repetitive region of unknown length within the non-coding control region. The mitogenome’s GC content is 31.80%. Phylogenetic analysis using protein-coding genes confirms the sister taxon relationship of *S. cafer* with the only other congener whose mitogenome has been reconstructed to date, the Asian *S. sirindhornae.*

Fairy shrimps (Anostraca) are freshwater crustaceans that occur exclusively in seasonally astatic aquatic habitats (Brendonck et al. 2008; Rogers 2009). Similar to other branchiopod members of the order, fairy shrimps produce dormant eggs which allow the survival of the next generation through dry periods (Brendonck et al. 2008; Rogers 2009) and demonstrate rapid body growth rates to facilitate attainment of sexual maturity within limited hydroperiod windows (Rogers 2009). The Anostraca originated in the Cambrian (Harvey et al. 2012), with the monobasic Streptocephalidae probably appearing during the early Cretaceous, approximately 105 million years ago (Daniels et al. 2004). The genus *Streptocephalus* has about 66 species worldwide, with numerous species occuring in southern Africa (Rogers 2013; Rogers and Padhye 2014; Shu et al. 2018). Despite their ecological significance, only a few complete mitogenomes of Anostracans have been published (Fan et al. 2015; Liu et al. 2016). Here, we describe the first complete genome of a widespread southern African fairy shrimp, *Streptocephalus cafer* (Lovén, 1847) (sensu Hamer et al. 1994).

Specimens of *S. cafer* were collected from a temporary pool on the outskirts of Palapye, Central District, Botswana (27.16616 E, 22.54507 S) and stored separately in 1.5 mL tubes containing 80% ethanol. Voucher specimens from the locality were lodged at the Kansas Biological Survey (DCR-1136). Total genomic DNA was extracted using the CTAB method (Doyle & Doyle 1987). One µg of DNA was used to prepare a genomic DNA library using the NEBNext DNA Library Preparation Kit (Massachusetts, USA). The library was then sequenced on an Illumina Hi-Seq platform using 2 × 150 chemistry with an average insert length of 350 bp.

The sequencing run yielded 59,848,500 paired-end sequences. The complete mitogenome was assembled using NOVOPlasty v3.5 (Dierckxsens et al. 2017) and annotated in MitoZ v.2.4 . The mitogenome assembly resulted in two contigs (15653 bp and 1347 bp, respectively) separated by a repetitive region of unknown length within the non-coding control region. MITOS annotation on the longest contig identified all 13 protein-coding genes, 22 tRNAs and 2 rRNAs, typical of crustaceans. The GC content of the total assembly was estimated at 31.8%. MitoZ annotation reported several instances of non-canonical start codons and truncated stop codons, consistent with other studies on arthropods (Monsanto et al. 2019).

Protein-coding sequences from *S. cafer* and the complete mitogenomes of nine related species were aligned in MAFFT v7.429 (Katoh et al. 2009). A Bayesian phylogenetic tree was reconstructed with BEAST2 (Bouckaert et al. 2014) using default parameters, except that the substitution model was changed to HKY (Hasegawa et al. 1985) with four gamma categories. BEAST2 was run for 50,000,000 iterations with 30% burn-in. The convergence of the chain and Effective Sample Size (ESS) was assessed in Tracer v1.7 (Rambaut et al. 2018), and the resulting phylogenetic tree was visualized in FigTree v1.4 (Rambaut and Drummond 2012) (Figure 1). It confirms the sister taxon relationship of *S. cafer* with the only other *Streptocephalus* species whose mitogenome has been reconstructed to date, the Asian *S. sirindhornae.*

**Figure 1.**
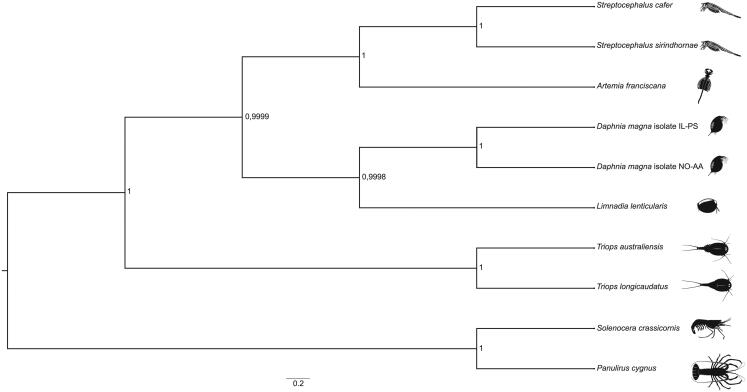
A Bayesian phylogenetic tree constructed in BEAST2 using mitogenome sequences of *Streptocephalus cafer* (NCBI accession number MN720104) and nine other crustacean species: *Streptocephalus sirindhornae* (NC_026704.1), *Daphnia magna* isolate IL-PS (MH683649.1), *Daphnia magna* isolate NO-AA(MH683655.1), *Artemia franciscana* (X69067.1), *Limnadia lenticularis* (NC_039394.1), *Panulirus cygnus* (KT696496.1), *Solenocera crassicornis* (KU899137.1), *Triops australiensis* (LK391946.1) and *Triops longicaudatus* (AY639934.1). The numbers next to each node represent posterior probability and the scale bar shows the scaled substitution rate.
